# Genotype-dependent responses of pepper endophytes to soil microbial community shifts induced by allyl isothiocyanate fumigation

**DOI:** 10.3389/fmicb.2025.1520443

**Published:** 2025-05-14

**Authors:** Wenfeng Tian, Qiuxia Wang, Aocheng Cao, Dongdong Yan, Yuan Li, Zhoubin Liu, Bozhi Yang, Wensheng Fang

**Affiliations:** ^1^Engineering Research Center for Horticultural Crop Germplasm Creation and New Variety Breeding, Ministry of Education, Key Laboratory of Vegetable Biology of Hunan Province, College of Horticulture, Hunan Agricultural University, Changsha, Hunan, China; ^2^State Key Laboratory for Biology of Plant Diseases and Insect Pests, Institute of Plant Protection, Chinese Academy of Agricultural Sciences, Beijing, China

**Keywords:** soil fumigation, endophyte, soil microorganism, AITC, genotype, pepper

## Abstract

**Introduction:**

Allyl isothiocyanate (AITC) has demonstrated efficacy as a soil fumigant, effectively controlling soil-borne pathogens and nematodes. Although AITC has a significant effect on soil microbial communities, whether fumigation affects the production of crop endophytes is unclear.

**Methods:**

In this study, AITC was used to fumigate the soil, and the response of endophytic bacteria (in roots, stems, and leaves) in different pepper genotypes (Xiangla359, La Xuan, Shuang Jiao) was investigated.

**Results:**

Fumigation with AITC significantly increased soil microbial diversity, stimulated the growth of Actinomycetota, and inhibited Pseudomonadota. However, the effects on endophytic bacteria varied among pepper varieties. Specifically, fumigation significantly reduced microbial diversity in the roots and leaves of Xiangla359, but had no significant effect on La Xuan and Shuang Jiao. Furthermore, the growth-promoting effect of AITC was most pronounced in Xiangla359.

**Conclusion:**

Our results suggest that while AITC fumigation significantly alters soil microbial diversity and composition, its effects on crop endophytes are genotype-dependent. These findings provide insight into the complex interactions between soil microbial communities and crop endophytes in response to soil fumigation.

## 1 Introduction

Peppers (*Capsicum annuum L.*), esteemed for their piquant flavor and robust nutritional profile, belong to the Solanaceae family and are renowned for their abundant polyphenols, capsaicinoids, ascorbic acid, and other phytochemicals (Barboza et al., 2022). In China, peppers are among the most extensively cultivated vegetables, with the 2021 cultivated area exceeding 213 ha, representing approximately 10% of the total vegetable cultivation acreage. The expansion of protected cultivation has driven a remarkable surge in vegetable yields; however, this progress has been paralleled by the accumulation of soil pathogens and exacerbation of soil-borne diseases, thereby impeding agricultural advancement (Ren et al., 2018). Consequently, addressing the persistent crop disorders in peppers and enhancing their yield constitute pivotal concerns in contemporary agricultural practices.

Soil fumigation represents the most direct and effective strategy for addressing the challenges associated with crop recolonization (Mao et al., 2014). Among the various fumigants, allyl isothiocyanate (AITC) stands out as a high-quality, plant-derived agent known for its efficacy in preventing and eradicating a range of soil-borne pathogenic nematodes (Dahlin and Hallmann, 2020; Ren et al., 2018), pathogenic fungi (Yu et al., 2019) and weeds (Zhang Y. et al., 2023). Additionally, AITC effectively inhibits pests during storage (Wu et al., 2009) and mitigates postharvest diseases (Otoni et al., 2014; Ugolini et al., 2014) all while maintaining a high safety profile and leaving no pesticide residues.

Recent studies have demonstrated that AITC significantly inhibits the development of gray mold in tomatoes, thereby extending their storage life (Barea-Ramos et al., 2024). Furthermore, AITC has been shown to effectively mitigate postharvest quality deterioration in edible mushrooms and raspberries (Park et al., 2023; Zou et al., 2024). Yu et al.’s (2022) research revealed that the inhibitory effect of AITC on C. heterostrophus was linked to the downregulation of genes associated with energy metabolism, oxidoreductase activity, melanin biosynthesis, and cell wall-degrading enzymes, resulting in a significant reduction in melanin production in C. heterostrophus. Moreover, AITC has been proven to effectively inhibit various pathogenic bacteria, including Rhizoctonia solani, Fusarium graminearum, Ralstonia solanacearum, P. aphanidermatum, Fusarium spp., and Phytophthora spp (Ashiq et al., 2022; Handiseni et al., 2017; Ren et al., 2018).

Soil microorganisms play a crucial role in environmental processes and are pivotal in maintaining the stability of soil ecosystems (Wu et al., 2024). The non-targeted nature of the fumigant led to a notable reduction in the population of detrimental soil microorganisms, such as Fusarium oxysporum and Mycobacterium avium. The application of AITC fumigation resulted in a transient decline in the overall microbial abundance in the soil, which gradually recovered over a one-month period (Castellano-Hinojosa et al., 2022). Furthermore, a significant increase in beneficial soil bacteria was observed following AITC fumigation (Tagele et al., 2021). AITC soil fumigation was also found to enhance the population of aerobic denitrifying bacteria (Li et al., 2023). Moreover, AITC fumigation and its degradation products demonstrated effective inhibition of soil manganese oxidation, leading to an increase in the bioavailable manganese content in the soil (Wang et al., 2024).

The diversity of inter-root microorganisms is intricately modulated by factors such as pH, effective phosphorus levels, and organic matter content (Zhou et al., 2021). Additionally, the species composition and abundance of the rhizosphere microbiome are influenced by the developmental stages of plants and the specific plant species present (Chen et al., 2022). Disparities in the distribution and abundance of inter-root and endophytic microorganisms significantly impact various physiological processes in plants, including growth regulation, disease resistance, and hormonal signaling pathways (Hartmann and Six, 2023; Yang et al., 2023). Endophytic microorganisms represent a category of microbes that engage in mutually beneficial symbiotic relationships with their host plants. Due to their prolonged coexistence within the unique plant environment, these microorganisms are recognized as valuable microbial resources that significantly contribute to the growth, development, and stress resilience of crops (Compant et al., 2019). Endophytes facilitate plant growth by providing essential nutrients or conferring protection against pathogenic challenges. The potential of plant endophytic microorganisms to enhance crop resistance and stimulate yield gains has attracted considerable research attention (Afzal et al., 2019; Sessitsch et al., 2012). Among these microorganisms, endophytic fungi have emerged as a focal point of investigation. Several studies have demonstrated that endophytic fungi play a significant role in enhancing crop nitrogen utilization (Yang et al., 2014). Notably, the presence of the endophytic bacterium Paenibacillus exerts a profound influence on the metabolic profile of isolated cultivated poplar plants (Scherling et al., 2009). Several bacterial and fungal genomes, such as those of Penicillium, Fusarium, Phytophthora, Dendrobacter, and *Streptomyces*, contain genes associated with nitrogen metabolism, hormone biosynthesis, phosphate utilization, and root colonization mechanisms (Huang et al., 2022; Shah et al., 2022). These Microorganisms play a crucial role in providing essential nutrients to plants, thereby facilitating growth promotion through mechanisms such as nitrogen fixation and enhanced access to phosphorus and iron (Liu et al., 2022; Phurailatpam et al., 2022).

Although the interactions between soil microbiota and crop endophytes are acknowledged to be crucial for plant health, the specific mechanisms underlying these interactions remain unclear. It has been demonstrated that soil microbial diversity and composition significantly impact plant growth and health, thereby influencing the dynamics of endophytic flora. However, changes in soil microbial communities do not invariably lead to shifts in crop endophytes, as the distinct habitats of endophytes are primarily shaped by the plant’s internal environment. Soil management practices, such as fertilization, microbial inoculation, and fumigation treatments, can modulate microbial diversity and abundance in the soil, subsequently influencing the microbial communities within the plant’s root system and above-ground structures, including endophyte populations. In this investigation, we employed HiSeq2500 high-throughput sequencing technology to explore variations in microbial community diversity and the structural composition of endophytes in different pepper varieties following AITC fumigation. Our study aims to elucidate the potential impact of changes in soil microbial communities on crop endophyte communities.

## 2 Materials and methods

### 2.1 Indoor soil fumigation experiment

The experimental soil used in this study was sourced from Yanqing, Beijing (115°44’–116°34’E, 40°16’–40°47’N) and is classified as alkaline tidal soil. Prior to use, the soil was sieved through a 2 mm mesh. A total of 12 kg of the test soil was weighed and placed into a 20 L plastic bucket. A 20% water emulsion of propylene isothiocyanate (AITC), obtained from Beijing Agronon Bio-Pharmaceutical Co., Ltd., was thoroughly mixed with the soil. Immediately after mixing, the bucket was sealed tightly with a transparent plastic cover and securely wrapped with transparent tape. The mixture was then incubated at 28°C in a light-free environment for a duration of 10 days. A control group without the addition of AITC was also included. Following the fumigation period, the soil was left uncovered in a well-ventilated area for one week to ensure the complete dispersion of any remaining fumigant. Subsequently, 30 g of the soil was collected and stored at −80°C for macrogenomic sequencing.

### 2.2 Pepper planting

The pepper seeds were pre-soaked and allowed to germinate for 3 days prior to sowing. Subsequently, the seeds were evenly distributed in the soil, covered with approximately 1 cm of loose soil, watered once, and maintained at a constant temperature of around 28°C. Regular watering was carried out to ensure soil moisture levels were optimal. Each experimental group consisted of 42 plants. The incubation room was set to a temperature of 28°C, with a relative humidity of 60% and a photoperiod of L//D = 12H//12H.

### 2.3 Sample collection

Sampling was conducted on the 55th day after pepper transplantation to assess plant height, stem thickness, and chlorophyll content. Chlorophyll levels were measured using a handheld chlorophyll meter. On the same day, rhizosphere soil samples were collected. During the sampling process, the peppers were carefully uprooted, and any loose soil attached to the root system was gently shaken off. Tightly adhered soil was then collected using a brush. A portion of the soil was stored at 4°C for the evaluation of physicochemical indices, while another portion was stored at −80°C for high-throughput sequencing.

The preparation of pepper samples involved washing the roots, stems, and leaves with distilled water to remove impurities. After washing, the roots, stems, and leaves were cut and soaked in 75% ethanol for 2 min, followed by a 5-min wash with a 2% sodium hypochlorite solution. Subsequently, the samples were transferred to 75% ethanol for 1 min and rinsed three times with sterile water, repeating this process three times. The samples were then dried, placed in self-sealing bags, and stored at −80°C for high-throughput sequencing (Ren et al., 2019). Each set of trials was conducted in triplicate.

### 2.4 Physicochemical properties of soil

Soil physicochemical parameters were analyzed following the method established by Li Sheik Kai. Nitrate nitrogen and ammonium nitrogen were extracted using KCl and quantified using a flow analyzer. Effective phosphorus was extracted with NaHCO_3_ and measured using a flow analyzer, while fast-acting potassium was extracted with CH_3_COONH_4_ and quantified using a flame spectrophotometer. pH was determined using a 2.5:1 water-to-soil ratio and potentiometric method, and electrical conductivity was measured using a 2.5:1 water-to-soil ratio and conductivity probe. Organic matter content was assessed through potassium dichromate titration.

### 2.5 Metagenomic sequencing

0.2 g of soil material was used to extract total genomic DNA with the E.Z.N.A.^®^ soil DNA Kit (Omega Bio-tek, Norcross, GA, USA) according to manufacturer’s instructions. Concentration and purity of extracted DNA was determined with SynergyHTX and NanoDrop2000, respectively. DNA quality was checked on 1% agarose gel.

DNA extract was fragmented to an average size of about 350 bp using Covaris M220 (Gene Company Limited, China) for paired-end library construction. Paired-end library was constructed using NEXTFLEX Rapid DNA-Seq (Bioo Scientific, Austin, TX, USA). Paired-end sequencing was performed on Illumina NovaSeq™ X Plus (Illumina Inc., San Diego, CA, USA) at Majorbio Bio-Pharm Technology Co., Ltd (Shanghai, China) using NovaSeq X Series 25B Reagent Kit according to the manufacturer’s instructions.^1^

### 2.6 DNA extraction, PCR amplification, and high-throughput sequencing

DNA extraction from treated plant samples was performed utilizing the Qiagen magnetic bead method, while total DNA from soil samples was isolated using the E.Z.N.A.^®^ Soil DNA Kit. Following extraction, the purity and concentration of DNA were evaluated using the Scandrop200 and confirmed by 1% agarose gel electrophoresis, respectively. Primers were tailed with PacBio barcode sequences to distinguish each sample. Amplification reactions (20-μL volume) consisted of 5 × FastPfu buffer 4 μL, 2.5 mM dNTPs 2 μL, forward primer (5 μM) 0.8 μL, reverse primer (5 μM) 0.8 μL, FastPfu DNA Polymerase 0.4 μL, template DNA 10 ng and DNase-free water. The PCR amplification was performed as follows: initial denaturation at 95°C for 3 min, followed by 27 cycles of denaturing at 95°C for 30 s, annealing at 60°C for 30 s and extension at 72°C for 45 s, and single extension at 72°C for 10 min, and end at 4°C (T100 Thermal Cycler PCR thermocycler, BIO-RAD, USA). After electrophoresis, The PCR products were purified using the AMPure^®^ PB beads (Pacifc Biosciences, CA, USA) and quantified with Qubit 4.0 (Thermo Fisher Scientific, USA). For amplification of the 16S rRNA gene in pepper root, stem, and leaf samples, primers 779F (AACMGGATTAGATACCCKG) and 1193R (ACGTCATCCCCACCTTCC) were employed, whereas primers 338F (ACTCCTACGGGAGGCAGCA) and 806R (GGACTACHVGGGTWTCTAAT) were utilized for soil samples. Subsequent high-throughput and macro-genome sequencing were conducted by Shanghai Meiji Biotechnology Co., Ltd.

### 2.7 Statistical analyses

The purified amplicons were pooled in equimolar and paired-end sequenced (2 × 300) on an Illumina MiSeq platform (Illumina, San Diego, USA). The original sequences were sequenced, spliced, quality controlled, and filtered to obtain optimized sequences (Magoc and Salzberg, 2011), clustered into operable taxonomic units (OTUs) according to similarity levels. The optimized sequences were compared with the Silva (SSU123)16S rRNA Database. A confidence threshold of 0.7 was chosen to combine with the RDP classifier Bayesian algorithm v2.21 to taxonomically analyze (Magoc and Salzberg, 2011), OTU sequences at 97% similarity level to obtain species annotation information for OTUs. The best-hit taxonomy of non-redundant genes was obtained by aligning them against the NCBI NR database by DIAMOND[6] (version 2.0.13)^2^ with an e-value cutoff of 1e-5. Similarly, the functional annotation (GO, KEGG, eggNOG, CAZy, CARD, PHI) of non-redundant genes was obtained. Based on the taxonomic and functional annotation and the abundance profile of non-redundant genes, the differential analysis was carried out at each taxonomic, functional, or gene-wise level by Kruskal–Wallis test. The experimental data was organized using Microsoft Excel 2010 software. The comparison of means between groups was conducted using SPSS 26 for independent samples *t*-test and one-way ANOVA. The significance level was set at α = 0.05. PcoA was generated using the Bray Curtis algorithm, and community composition and variability analyses were performed using the Meguiar’s Cloud platform. Graphing was carried out using Origin 2017. The Welch *t*-test method was used to calculate the confidence interval. The stats package (version 3.3.1) of R and the scipy package (version 1.0.0) of Python were used. The linear discriminant analysis (LDA) effect size (LEfSe)^3^ was performed to identify the significantly abundant taxa (phylum to genera) of bacteria among the different groups (LDA score > 3.8, *P* < 0.05).

## 3 Results

### 3.1 Effects of allyl isothiocyanate on soil microorganisms and functions

Gene-based species taxonomic annotations were compared with the NR database to extract species and abundance information across various taxonomic levels, including domain, kingdom, phylum, order, family, genus, and species for each sample. A total of 244 phyla, 501 orders, 1,003 families, 1,966 genera, and 30,841 species were identified through NR species annotation. The results of the bacterial community alpha diversity index (Table 1) demonstrate that AITC fumigation significantly enhanced the richness of the soil microbial community.

**TABLE 1:** Alpha diversity index of soil microbial community fumigated by allyl isothiocyanate.

Sample	Ace	Shannon	Simpson
CK	8.5 ± 0.50	1.95 ± 0.002	0.14 ± 0.009
AITC	12.33 ± 0.67	2.30 ± 0.08	0.11 ± 0.005

**FIGURE 1:**
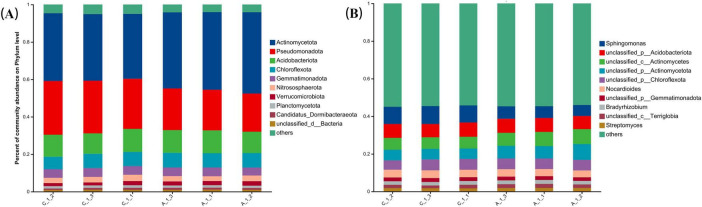
Bar chart showing changes in the relative abundance of the top ten bacterial communities at the phylum level **(A)** and genus level **(B)** in soil samples.

**FIGURE 2:**
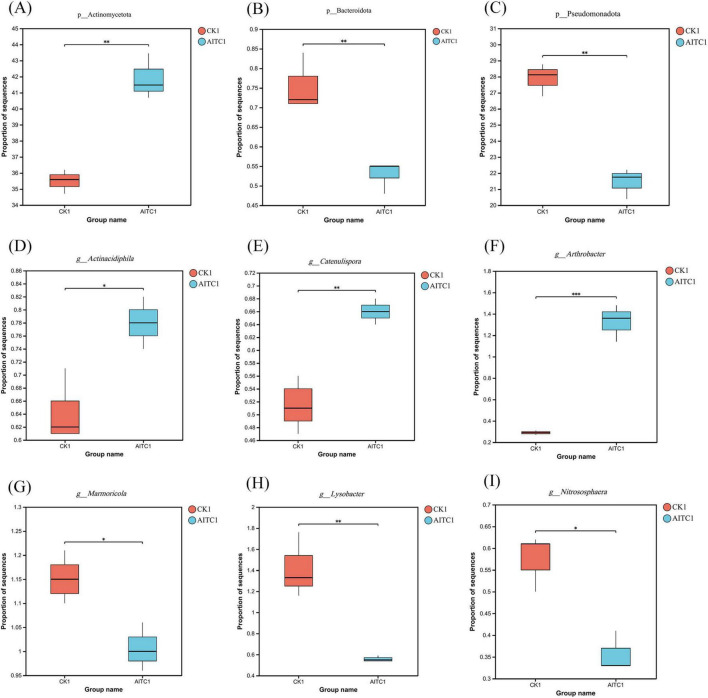
Microorganisms with significant differences in soil microorganisms at the phylum level **(A–C)** and genus level **(D–I)** after AITC fumigation. *0.01 < *p* ≤ 0.05, **0.001 < *p* ≤ 0.01.

A comparative analysis of soil microorganisms before and after allyl isothiocyanate (AITC) fumigation revealed shifts in the top ten microorganisms at the phylum level within the soil microbial community (Figure 1A). The predominant phyla identified were Actinomycetota, Pseudomonadota, Acidobacteriota, Chloroflexota, Gemmatimonadota, Nitrososphaerota, Verrucomicrobiota, Planctomycetota, Candidatus Dormibacteraeota, and unclassified d_Bacteria. Notably, the relative abundance of Actinomycetota showed a significant increase (*P* < 0.01) post-fumigation. Conversely, there was a marked decrease in the relative abundance of Pseudomonadota and Bacteroidota (*P* < 0.01), indicating that Actinomycetota exhibited a significant increase (*P* < 0.01) (Figures 2A–C).

At the genus level (Figure 1B), the top ten microorganisms in relative abundance in the soil microbial community were *Sphingomonas*, *unclassified_ p_Acidobacteriota*, *unclassified_c_Actinomycetes*, *unclassified_p_ Actinomycetota*, *unclassified_p_Chloroflexota*, *Nocardioides*, *unclassified_p_Gemmatimonadota*, *Bradyrhizobium*, *unclassified_ c_Terriglobia*, *Streptomyces*. Of which, *Sphingomonas*, *Lysobacter*, *Nitrososphaera*, *Marmoricola*, *Candidatus_Nitrosocosmicus*, *Phycicoccus*, *Nitrosopumilus* showed significant reduction in relative abundance after fumigation, *Arthrobacter*, *Blastococcus*, *Pseudarthrobacter*, *Actinacidiphila*, *Catenulispora*, *Mycobacterium*, *Pseudonocardia*, and *Trebonia* increased significantly in relative abundance (Figures 2D–I).

**FIGURE 3:**
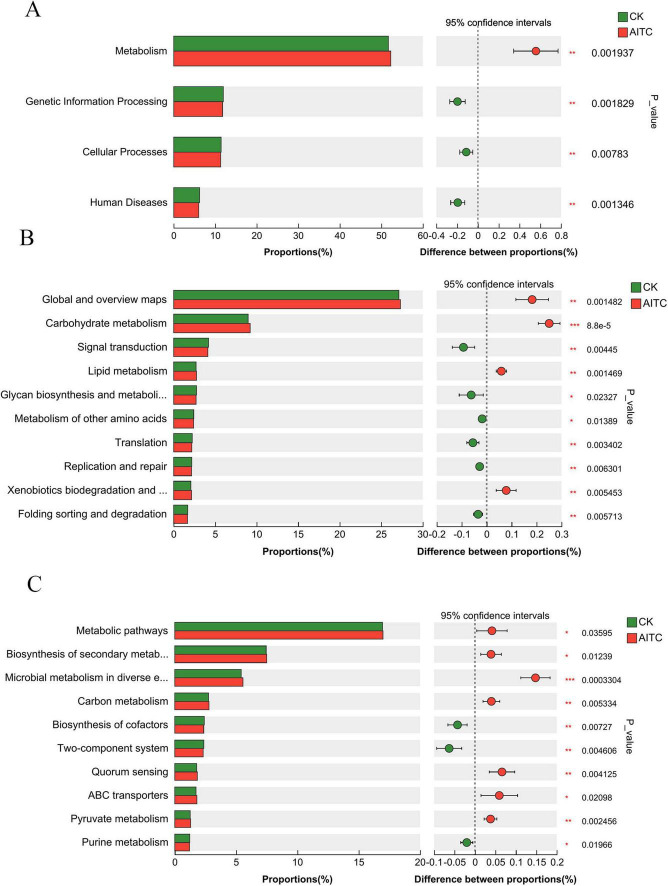
The KEGG functional levels of soil microorganisms after AITC fumigation were significantly different. **(A)** Pathway level 1. **(B)** Pathway level 2. **(C)** Pathway level 3.

By comparing the results with the KEGG database, the top three annotations at Level 1 (Figure 3A) in soil samples were identified as Metabolism, Environmental Information Processing, and Genetic Information Processing. The known gene functions with the top 10 abundances were analyzed using ANOVA. The analysis of variance revealed that the Metabolism function was significantly higher in fumigated soil compared to unfumigated soil (*P* < 0.05), while Genetic Information Processing, Cellular Processes, and Human Diseases were significantly lower in the fumigated soil (*P* < 0.05). At Level 2 (Figure 3B), metabolic pathways were also significantly higher in the soil samples than in the unfumigated soil, with significant increases observed in the following pathways: Biosynthesis of Secondary Metabolites, Microbial Metabolism in Diverse Environments, Carbon Metabolism, Quorum Sensing, ABC Transporters, and Pyruvate Metabolism (*P* < 0.05). The top 10 pathways annotated at the Pathway classification level that exhibited significant differences (Figure 3C) included Biosynthesis of Secondary Metabolites, Microbial Metabolism in Diverse Environments, Carbon Metabolism, Biosynthesis of Cofactors, Two-Component System, Quorum Sensing, ABC Transporters, Pyruvate Metabolism, and Purine Metabolism.

### 3.2 Effects of allyl isothiocyanate on the growth of peppers and soil physicochemical indexes

**FIGURE 4:**
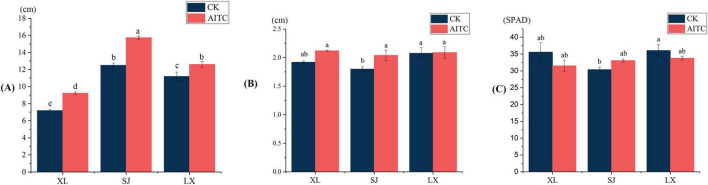
The impact of AITC fumigation on the growth of pepper plants. **(A)** Height of pepper plants under different treatments. **(B)** Stem diameter of chili pepper plants under different treatments. **(C)** Chlorophyll content of chili pepper leaves under different treatments. All results are expressed as mean ± standard deviation (SD), and the same letters indicate no statistical difference (*P* = 0.05) according to Duncan’s new multiple range test.

Allyl isothiocyanate (AITC) fumigation exerts a positive effect on the growth and development of peppers, although varietal discrepancies are evident (Figure 4). By the 55th day post-planting, fumigation significantly enhanced the plant height of Xiangla359 and Shuang Jiao, as well as the stem thickness of Shuang Jiao. Specifically, in the treatment group, the plant height of Xiangla359 exhibited a significant increase of 28.2%, accompanied by a notable 10.4% increase in stem thickness. Moreover, La Xuan demonstrated a growth of 12.5% in plant height, while Shuang Jiao exhibited a substantial rise of 25.9%. As depicted in Table 2, the impacts of soil fumigation with AITC on the physicochemical indicators of the rhizosphere soil for various chili pepper cultivars varied; however, these variations were not deemed statistically significant.

**TABLE 2:** Effect of soil fumigation with allyl isothiocyanate on physicochemical indexes of rhizosphere soil of different varieties of capsicum.

	Conductivity (μs/cm)	pH	Organic matter (mg/kg)	Available *P* (mg/kg)	Available K (mg/kg)	NH_4_^+^-N (mg/kg)	NO_3_^–^-N (mg/kg)
CK-XL	252.33 ± 20.36a	7.88 ± 0.11a	25.01 ± 1.30a	334.36 ± 4.88a	260 ± 6.62b	19.69 ± 13.67a	23.73 ± 0.01a
AI-XL	297.33 ± 11.88a	7.66 ± 0.03a	26.39 ± 1.16a	350.88 ± 4.63a	335 ± 42.57ab	16.70 ± 4.70a	23.75 ± 0.03a
CKLX	250.33 ± 18.13a	7.61 ± 0.005a	26.88 ± 0.79a	353.62 ± 3.43a	284.17 ± 34.69ab	20.16 ± 9.79a	23.77 ± 0.04a
AI-LX	245 ± 25.97a	7.55 ± 0.02a	25.72 ± 1.27a	444.92 ± 96.79a	260.83 ± 3.34b	31.91 ± 15.54a	23.75 ± 0.04a
CK-SJ	306.33 ± 27.77a	7.78 ± 0.005a	25.94 ± 0.44a	349.89 ± 12.30a	360 ± 31.26ab	16.70 ± 5.09a	24.33 ± 0.62a
AI-SJ	278 ± 17.69a	7.62 ± 0.07a	26.74 ± 0.11a	373.51 ± 5.35a	381.67 ± 52.19a	10.95 ± 2.63a	23.71 ± 0.001a

All results are expressed as mean ± standard deviation (SD), and the same letters indicate no statistical difference (*P* = 0.05) according to Duncan’s new multiple range test.

### 3.3 Effect of soil fumigation with allyl isothiocyanate on the diversity index of the bacterial community of pepper

**FIGURE 5:**
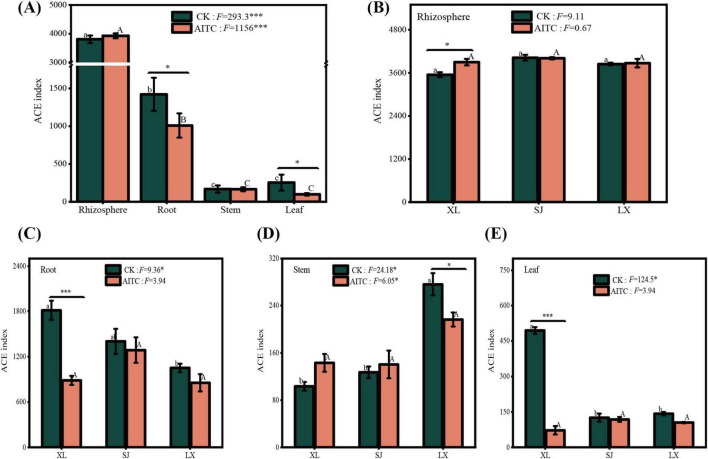
The impact of AITC fumigation on the microbial diversity index of chili plants: **(A)** overall effect on different tissues; **(B)** response of rhizosphere bacteria; **(C)** response of endophytic fungi in roots; **(D)** response of endophytic bacteria in stems; **(E)** response of endophytic bacteria in leaves. *0.01 < *p* ≤ 0.05, ***p ≤ 0.001.

Significant variations in the bacterial community diversity index (ACE) were observed among different parts of the pepper plant, including inter-root, intra-root, intra-stem, and intra-leaf regions, regardless of the fumigation treatment (Figure 5A). These findings suggest that tissue variability remained consistent even after fumigation. However, 80 days post-fumigation, the ACE values for intra-root and intra-leaf bacterial diversity were significantly reduced by allyl isothiocyanate (AITC), while no significant impact was observed on inter-root and intra-stem bacterial diversity. This indicates that the stimulating effect of AITC on soil microbial community diversity diminished over time but continued to significantly influence microbial diversity within the roots and leaves of the crop.

Further analysis of the responses of bacterial communities in the root, stem, and leaf tissues of three pepper varieties revealed distinct spatial characteristics and significant differences in the effects of allyl isothiocyanate (AITC) fumigation on endophytic bacteria. Inter-root bacterial diversity showed no significant differences among Xiangla359 (XL), Shuang Jiao (SJ), and La Xuan (LX). However, AITC fumigation significantly increased the inter-root bacterial diversity index (ACE) in Xiangla359 (Figure 5B). Within the roots, both Xiangla359 and Shuang Jiao exhibited significantly higher bacterial diversity compared to La Xuan. Nevertheless, AITC fumigation resulted in a significant decrease in bacterial diversity within the roots of Xiangla359, with no significant differences observed among the three varieties after fumigation (Figure 5C). Conversely, La Xuan displayed significantly higher bacterial diversity in the stems compared to Xiangla359 and Shuang Jiao. However, AITC fumigation led to a significant reduction in bacterial diversity in the stems of La Xuan, with no significant differences among the three varieties after fumigation (Figure 5D). In terms of leaf tissues, Xiangla359 exhibited significantly higher bacterial diversity compared to Shuang Jiao and La Xuan. AITC fumigation significantly reduced bacterial diversity in the leaves of Xiangla359 but had no significant effect on Shuang Jiao and La Xuan (Figure 5E). Overall, the impact of AITC fumigation on endophytes in pepper roots and leaves was more pronounced than in stems, resulting in a significant reduction in microbial diversity in the roots and leaves of Xiangla359, while showing no significant effects on Shuang Jiao and La Xuan.

### 3.4 Effect of allyl isothiocyanate on the structure of the bacterial community of chili peppers

**FIGURE 6:**
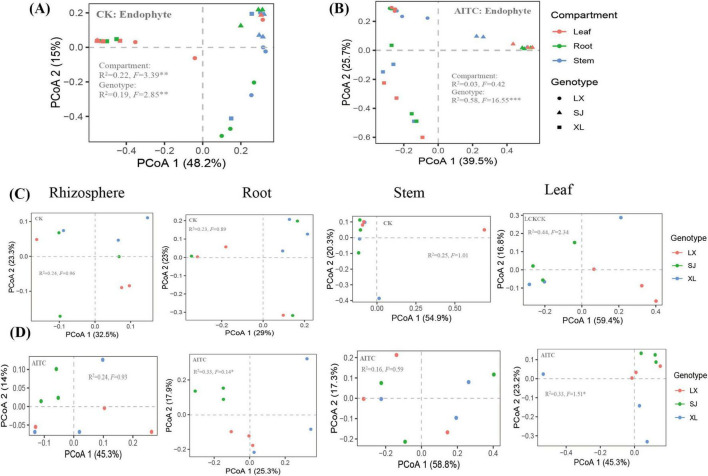
Based on the Bray Curtis algorithm PCoA (Principal Co-ordinates Analysis), **(A)** PCoA of endophytic bacteria in untreated samples; **(B)** PCoA of endophytic bacteria treated with AITC (Allyl isothiocyanate); **(C)** PCoA of rhizosphere, endophytic, stem, and leaf bacteria in untreated samples; **(D)** PCoA of rhizosphere, endophytic, stem, and leaf bacteria in treated samples. PERMANOVA method was used to calculate statistical differences.

In the unfumigated treatment, both tissue site and genotype significantly influenced the structure of the endophytic bacterial community in pepper, accounting for 22% (*p* < 0.01) and 19% (*p* < 0.01) of the variation, respectively (Figure 6A). Conversely, allyl isothiocyanate (AITC) fumigation markedly enhanced the contribution of genotype to the endophytic bacterial community structure in pepper, increasing it to 58% (*p* < 0.001) while concurrently reducing the contribution of tissue site (Figure 6B).

Consequently, we conducted a detailed analysis of the bacterial community structure across various tissues (roots, stems, and leaves) of different pepper cultivars. Following to AITC fumigation, the impact of genotypes on inter-root and intra-stem bacterial community structures was found to be statistically insignificant. However, there was a notable increase in the explanatory power of the bacterial community structure in roots (23 vs. 33%, *p* < 0.05), accompanied by a corresponding decrease in leaves (44 vs. 33%, *p* < 0.05) (Figures 6C, D). It is evident that allyl isothiocyanate (AITC) fumigation primarily influences the bacterial community structure in roots and leaves of pepper and is closely associated with the genotype of the plant.

**FIGURE 7:**
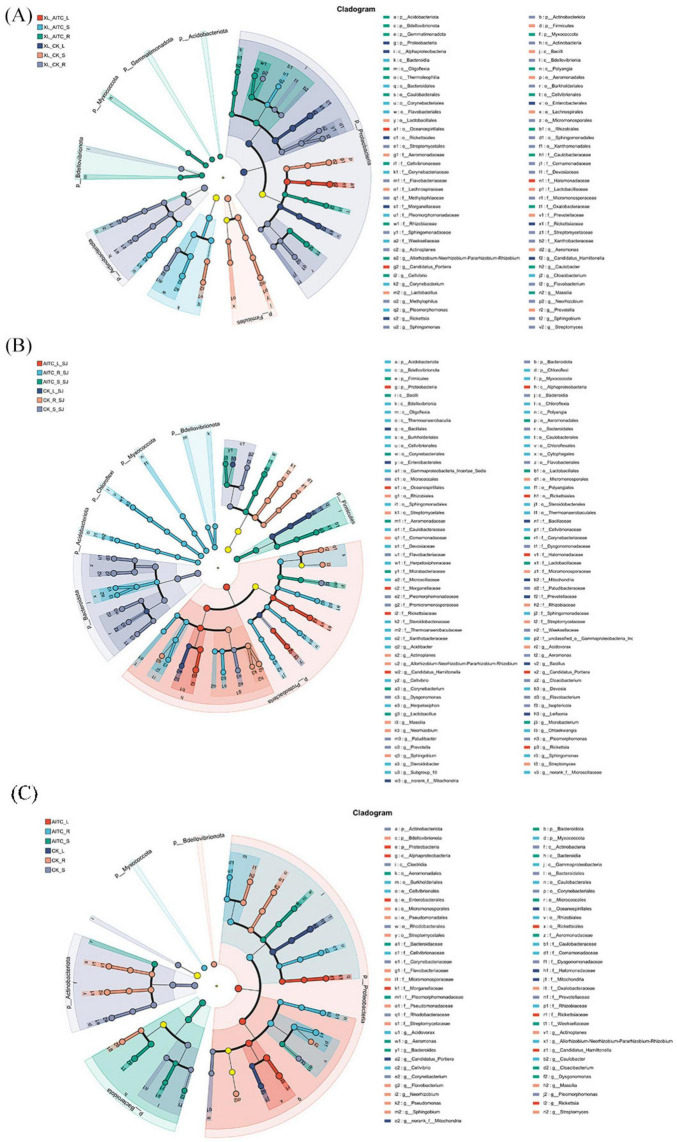
LEfSe cladogram analysis of endophytic microbial community (LDA = 3.8) in pepper after soil fumigation (*P* = 0.05). The five rings in the cladogram represent the phylum, class, order, family and genus, respectively, when read from the inside. The different color nodes (except yellow) on the ring represent significant changes in taxonomic composition due to the treatments. **(A)** Xiangla359; **(B)** Shuangjiao; **(C)** Laxuan.

Linear discriminant analysis effect size (LEfSe) was employed to identify species with significant differences across various parts of pepper plants. The LEfSe species hierarchy chart (LDA > 3.8) was used to identify groups exhibiting significant variations. As shown in the distribution plots (Figures 7A–C) with LDA scores exceeding 3.8, significant differences were observed in the microbial communities enriched in different parts of the pepper plant under various treatments.

At the genus level, untreated Xiangla359 roots exhibited a marked predominance of *Streptomyces*, *Sphingomonas*, *Sphingobium*, *Methylophilus*, *Neorhizobium*, and *Flavobacterium*. In contrast, the stems showed a significant increase in Prevotella, *Lactobacillus*, and *Aeromonas*, while the leaves were predominantly dominated by *Rickettsia*. Following AITC treatment, the roots displayed a significant abundance of *Cellvibrio*, *Massilia*, *Caulobacter*, and *Allorhizobium-Neorhizobium-Pararhizobium-Rhizobium*. In the stems, *Pleomorphomonas*, *Corynebacterium*, and *Cloacibacterium* were notably abundant.

Untreated Shuang Jiao roots demonstrated substantial concentrations of *Streptomyces*, *Sphingobium*, *Neorhizobium*, *Massilia*, *Acidovorax*, and *Allorhizobium-Neorhizobium-Pararhizobium-Rhizobium*. The stems were notably enriched with *Actinoplanes*, *Prevotella*, *Paludibacter*, *Pleomorphomonas*, *Aeromonas*, *Cloacibacterium*, *Dysgonomonas*, and *Flavobacterium*, while *Bacillus* and *Leifsonia* were primarily concentrated in the leaves. After AITC treatment, the roots of Shuang Jiao displayed a notable rise in *Subgroup_10*, *Steroidobacter*, *Sphingomonas*, *Ohtaekwangia*, *Herpetosiphon*, *Cellvibrio*, *Acetobacter*, and *Devosia*. In the stems, *Corynebacterium*, *Lactobacillus*, and *Microbacterium* were significantly more abundant.

In untreated La Xuan roots, *Streptomyces*, *Sphingomonas*, *Sphingobium*, *Pseudomonas*, *Neorhizobium*, *Flavobacterium*, *Massilia*, and *Actinoplanes* were notably abundant. The stems exhibited significant accumulation of *Corynebacterium* and *Pleomorphomonas*. Following AITC treatment, the roots displayed a marked increase in *Cellvibrio*, *Acidovorax*, *Caulobacter*, and *Allorhizobium-Neorhizobium-Pararhizobium-Rhizobium*. In the stems, *Dysgonomonas*, *Aeromonas*, *Cloacibacterium*, and *Bacteroidota* were significantly more prevalent, while Rickettsia was predominantly concentrated in the leaves.

In the rhizosphere of pepper plants, the predominant bacterial phyla included *Actinobacteriota*, *Proteobacteria*, *Acidobacteriota*, *Chloroflexi*, *Firmicutes*, *Gemmatimonadota*, *Bacteroidota*, and *Myxococcota* (Supplementary Figure 1A). Fumigation with allyl isothiocyanate (AITC) significantly increased the relative abundance of *Armatimonadota* and enhanced the presence of *Acidobacteriota* in the inter-root region of Shuang Jiao. At the genus level, the primary taxa identified included *Streptomyces*, *Allorhizobium-Neorhizobium-Pararhizobium-Rhizobium*, *Pseudomonas*, *Massilia*, *Flavobacterium*, *Actinoplanes*, *Methylophilus*, and *Sphingobium* (Supplementary Figure 1B). Moreover, fumigation led to a substantial rise in the relative abundance of *Pseudarthrobacter* (34.21%) and *RB41* (62.21%), while reducing the abundance of *Gaiella* (41.09%) in the inter-root soil of Shuang Jiao.

**FIGURE 8:**
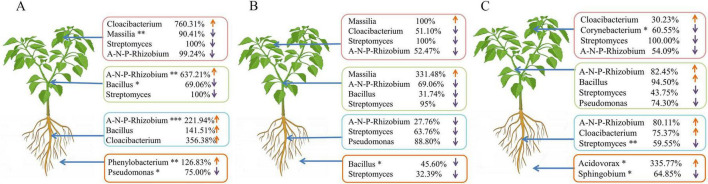
Changes in microorganisms in the roots, stems, leaves, and rhizosphere soil of different varieties of pepper plants. **(A)** Xiangla359; **(B)** Shuang Jiao; **(C)** La Xuan; A-N-P-Rhizobium: *Allorhizobium-Neorhizobium-Pararhizobium-Rhizobium* (*p < 0.05, ** < 0.01, *** < 0.001).

In the root systems of pepper plants, the predominant bacterial phyla identified were *Proteobacteria*, *Actinobacteriota*, *Firmicutes*, *Bacteroidota*, *Myxococcota*, *Bdellovibrionota*, *Chloroflexi*, *Acidobacteriota*, *Gemmatimonadota*, and *Verrucomicrobiota* (Supplementary Figure 2A). Fumigation with AITC significantly increased the relative abundance of *Myxococcota*, while simultaneously decreasing *Actinobacteriota* in the roots of Shuang Jiao. At the genus level, the major taxa present in pepper roots included *Streptomyces*, *Allorhizobium-Neorhizobium-Pararhizobium-Rhizobium*, *Pseudomonas*, *unclassified_f_Alcaligenaceae*, *Massilia*, *Flavobacterium*, *Actinoplanes*, *Methylophilus*, *Sphingobium*, and *unclassified_f_Oxalobacteraceae* (Supplementary Figure 2B). AITC fumigation notably enhanced the relative abundance of *Cloacibacterium* (356.38%) and led to significant increases in *Allorhizobium-Neorhizobium-Pararhizobium-Rhizobium* (221.94%), *Bacillus* (141.51%), *Pseudomonas* (191.47%), and *Corynebacterium* (209.91%) within the roots of Xiangla359. In contrast, fumigation reduced the abundance of *Cloacibacterium* (61.78%), *Streptomyces* (63.76%), *Pseudomonas* (88.80%), and *Massilia* (70.26%) in Shuang Jiao roots, while increasing that of *Corynebacterium* (148.74%) and *Bacillus* (13.88%). Similarly, in La Xuan roots, fumigation led to an increase in the relative abundance of *Cloacibacterium* (75.37%), *Allorhizobium-Neorhizobium-Pararhizobium-Rhizobium* (80.11%), and *Corynebacterium* (23.56%), while reducing *Streptomyces* (59.55%) and *Massilia* (40.36%) (Figure 8).

In pepper stems, the predominant bacterial phyla identified included *Proteobacteria*, *Firmicutes*, *Bacteroidota*, *Actinobacteriota*, *Deinococcota*, *Cyanobacteria*, *Gemmatimonadota*, *Myxococcota*, *Bdellovibrionota*, and *Verrucomicrobiota* (Supplementary Figure 3A). At the genus level, the dominant taxa were *Cloacibacterium*, *Corynebacterium*, *Bacillus*, *Aeromonas*, *Lactobacillus*, *Flavobacterium*, *Paludibacter*, *Prevotella*, and the *Allorhizobium-Neorhizobium-Pararhizobium-Rhizobium* group (Supplementary Figure 3B). Following fumigation, the *Allorhizobium-Neorhizobium-Pararhizobium-Rhizobium* group in Xiangla359 stems showed a significant increase in relative abundance (637.21%), accompanied by a 50.10% increase in *Corynebacterium*. In contrast, *Massilia* and *Pseudomonas* saw a decline in relative abundance by 63.85% and 24.61%, respectively. In the La Xuan stem microbiome, fumigation resulted in increased abundances of *Cloacibacterium* (153.46%), *Corynebacterium* (60.77%), *Bacillus* (94.50%), and *Allorhizobium-Neorhizobium-Pararhizobium-Rhizobium* (82.45%). Meanwhile, *Massilia*, *Pseudomonas*, and *Streptomyces* decreased by 94.50, 74.30, and 43.75%, respectively. In another cultivar, fumigation led to a 331.48% increase in *Massilia*, while relative abundances of *Cloacibacterium*, *Allorhizobium-Neorhizobium-Pararhizobium-Rhizobium*, and *Streptomyces* decreased by 38.02, 69.06, and 95%, respectively, within the stem microbiome (Figure 8).

In pepper leaves, the predominant bacterial phyla identified included *Proteobacteria*, *Firmicutes*, *Bacteroidota*, *Actinobacteriota*, *Deinococcota*, *Cyanobacteria*, *Gemmatimonadota*, *Myxococcota*, *Bdellovibrionota*, and *Verrucomicrobiota* (Supplementary Figure 4A). At the genus level, *Bacillus*, *Cloacibacterium*, *Corynebacterium*, *Prevotella*, and *Lactobacillus* were the most abundant genera (Supplementary Figure 4B). Fumigation with allyl isothiocyanate (AITC) induced notable shifts in these microbial communities, particularly increasing the relative abundance of *Cloacibacterium* (760.31%) in Xiangla359 leaves, while significantly decreasing *Massilia* (90.41%), *Pseudomonas* (48.43%), *Streptomyces* (100%), and *Allorhizobium-Neorhizobium-Pararhizobium-Rhizobium* (99.24%). In Shuang Jiao leaves, AITC treatment led to a 100% increase in *Massilia*, while *Corynebacterium* (52.47%), *Streptomyces* (100%), *Bacillus* (73.76%), and the *Allorhizobium-Neorhizobium-Pararhizobium-Rhizobium* group (52.47%) were reduced. Similarly, in La Xuan leaves, AITC exposure resulted in substantial increases in *Massilia* (1,209.09%) and *Pseudomonas* (144.73%), alongside reductions in *Corynebacterium* (60.55%), *Streptomyces* (100%), *Bacillus* (55.90%), and the *Allorhizobium-Neorhizobium-Pararhizobium-Rhizobium* group (54.09%) (Figure 8).

**FIGURE 9:**
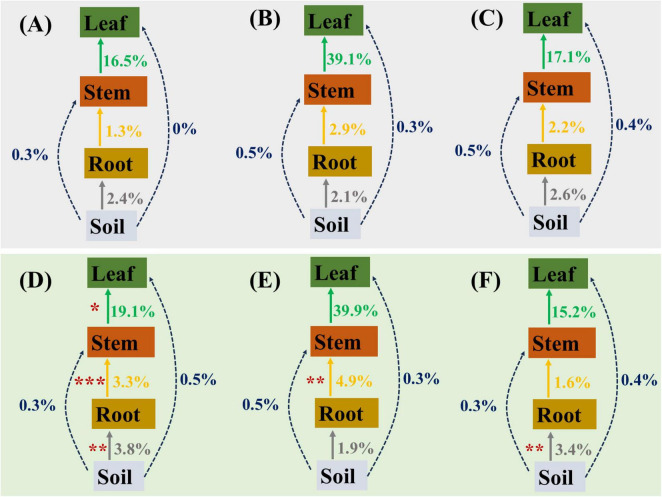
Traceability analysis of microorganisms in different genotypes of pepper across various sub-chambers (soil, root, stem, leaf) in the unfumigated **(A,B)** and AITC fumigated **(D–F)** groups. **(A,D)**: Xiangla359; **(B,E)**: Laxuan; **(C,F)**: Shuang Jiao. The values to the right of the arrows represent the percentage from source to sink, and the asterisk to the left of the arrows indicates a significant difference between the fumigated and control groups.

The traceability analysis results revealed that AITC fumigation significantly altered the distribution of the source and sink of the pepper microbiome across sub-compartments (soil, roots, stems, and leaves), particularly in Xiangla359 varieties. Compared to the non-fumigated group, the fumigation treatment promoted the accumulation of microorganisms in the roots, stems, and leaves of Xiangla359 peppers, while enhancing the enrichment of endophytic bacteria in the stems and roots of Laxuan pepper and Shuangjiao pepper respectively. This shift indicated that fumigation not only influenced the overall structure of the microbial community but also regulated the dynamic balance between source and sink by altering the migration and distribution of microorganisms. However, the extent of this regulation varied across genotypes (Figure 9).

## 4 Discussion

The microbial community structure in the rhizosphere of peppers shows considerable variability, with a notable degree of specificity in the interaction between plant genotypes and microbial composition (Weinert et al., 2011). Certain plant genotypes can selectively accumulate beneficial microorganisms, thereby enhancing plant defenses against pathogens (Berendsen et al., 2012). In this study, we observed significant differences in bacterial community diversity across different regions of the pepper plant, including inter-root, intra-root, intra-stem, and intra-leaf areas, independent of fumigation treatments. Furthermore, the variability in microbial composition within these tissues remained stable, irrespective of fumigation. Distinct plant genotypes appear to promote specific microbial enrichments, likely influenced by root-secreted compounds, inter-root environmental conditions, and additional biochemical factors (Cordovez et al., 2019). Moreover, AITC fumigation significantly altered soil metabolic processes, leading to changes in root distribution and inter-root exudation patterns.

The modification of soil microbial community structure by AITC fumigation influences microbial composition within the roots, stems, and leaves of peppers through various mechanisms: (1) Nutrient availability: AITC fumigation alters soil nutrient cycling, particularly by enhancing nitrogen and phosphorus mineralization, which significantly increases the availability of these nutrients for plant uptake (Fang et al., 2022; Huang et al., 2020; Li et al., 2023). Additionally, nutrient release from organic matter following fumigation—likely due to the suppression of specific microbial taxa—supports root nourishment. Compounds generated through these processes act as nutrient sources for soil microorganisms, selectively promoting the growth of particular microbial populations in the rhizosphere. This selective enrichment subsequently affects microbial community composition within the plant’s stems and leaves. (2) Microbial interaction: Interactions among soil microorganisms are intricate, involving both competitive and symbiotic relationships. Beneficial microorganisms are essential for enhancing plant resistance to pathogens, while harmful microorganisms can detrimentally impact plant health. The balance of these interactions strongly influences the microbial community composition within plant tissues. For example, allyl isothiocyanate (AITC) fumigation has been shown to increase soil microbial diversity and promote the growth of beneficial bacteria, such as Bacillus species. These beneficial microorganisms can colonize plant roots from the soil and subsequently move through the vascular system to the stems and leaves. This translocation process introduces novel microbial species and reshapes the existing microbial communities across different plant tissues. (3) Soil health: Prior to fumigation, soils generally exhibit low quality, often characterized by nutrient imbalances, degraded soil structure, and an unbalanced microbial community. Fumigation can significantly improve soil health by enhancing nutrient cycling and restructuring the microbial community (Cordovez et al., 2019). Healthy soils support high microbial diversity, which promotes vigorous plant growth. This robust plant growth stimulates the development of an extensive root system, thereby sustaining a diverse array of microorganisms within the roots and influencing microbial communities in the stems and leaves.

Fumigation with allyl isothiocyanate (AITC) induces significant shifts in soil microbial communities and functions, subsequently affecting the populations of beneficial bacteria in the roots, stems, and leaves of various pepper cultivars. AITC fumigation promotes the proliferation of diverse beneficial microorganisms, each with specialized biological functions. For example, *Allorhizobium-Neorhizobium-Pararhizobium-Rhizobium* species and nitrogen-fixing (Guo et al., 2024; Zhang W. et al., 2023), and *Streptomyces* strains play crucial roles in enhancing plant nutrient uptake (Olenska et al., 2020). Additionally, plant growth-promoting rhizobacteria (PGPR) such as *Pseudomonas* and the inter-root probiotic *Massilia* contribute to increased plant vigor and resilience (He et al., 2024). Cellulolytic activities are associated with *Cellvibrio* and *Sphingomonas*, while *Devosia* species are linked to inter-root exudates (Chen et al., 2019).

## 5 Conclusion

In this study, we investigated the effects of allyl isothiocyanate (AITC) soil fumigation on the endophytic bacterial communities associated with different pepper genotypes (Xiangla359, La Xuan, and Shuang Jiao). Our findings revealed that AITC fumigation predominantly influenced bacterial community structure in both roots and leaves, with a strong correlation to the genetic characteristics of the pepper cultivars. Notably, AITC fumigation led to a significant reduction in microbial diversity within the roots and leaves of Xiangla359, while having no substantial impact on La Xuan and Shuang Jiao.

Moreover, the growth-promoting effects of AITC fumigation varied among the three pepper varieties, with Xiangla359 exhibiting the most pronounced enhancement. This variation appears to be attributable to differential fumigation effects on beneficial bacterial populations across the roots, stems, and leaves of each cultivar. For instance, the *Allorhizobium-Neorhizobium-Pararhizobium-Rhizobium* increased in the roots and stems of Xiangla359 and La Xuan but decreased in Shuang Jiao. Similarly, *Bacillus* populations increased in the roots of Xiangla359 but declined in La Xuan and Shuang Jiao, while *Streptomyces* consistently decreased in the roots across all pepper varieties.

These results indicate that AITC fumigation significantly alters the diversity and structural composition of soil microbial communities; however, its effects on endophytic communities in pepper plants are highly dependent on the plant genotype.

## Data Availability

The raw sequence data reported in this paper have been deposited in the Genome Sequence Archive (Genomics, Proteomics & Bioinformatics 2021) in National Genomics Data Center (Nucleic Acids Res 2022), China National Center for Bioinformation / Beijing Institute of Genomics, Chinese Academy of Sciences (GSA: CRA025577) that are publicly accessible at https://ngdc.cncb.ac.cn/gsa.

## Footnotes

1
www.illumina.com

2
http://ab.inf.uni-tuebingen.de/software/diamond/

3
http://huttenhower.sph.harvard.edu/lefse
