# Supplementary material for: Genotype-dependent responses of pepper endophytes to soil microbial community shifts induced by allyl isothiocyanate fumigation
Source: Front Microbiol. 2025 May 14;16:1520443. doi: 10.3389/fmicb.2025.1520443 (PMC12120850; doi:10.3389/fmicb.2025.1520443)
Supplement: Supplementary file 1 [file Data_Sheet_1.docx]

Supplementary Material

## Supplementary Figures

**
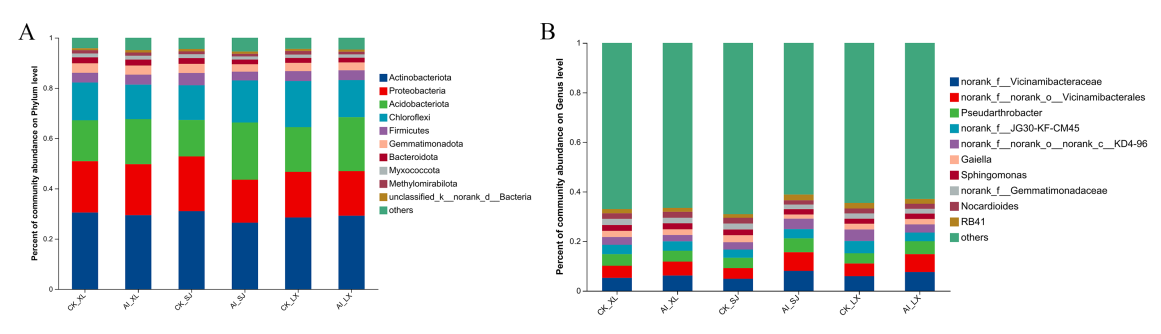
**

**Supplementary Figure 1.** The figure legends are required to have the same font as the main text, 12 point normal Times New Roman, single spaced. Please use a single paragraph for each legend and prepare the figures keeping in mind the PDF layout.


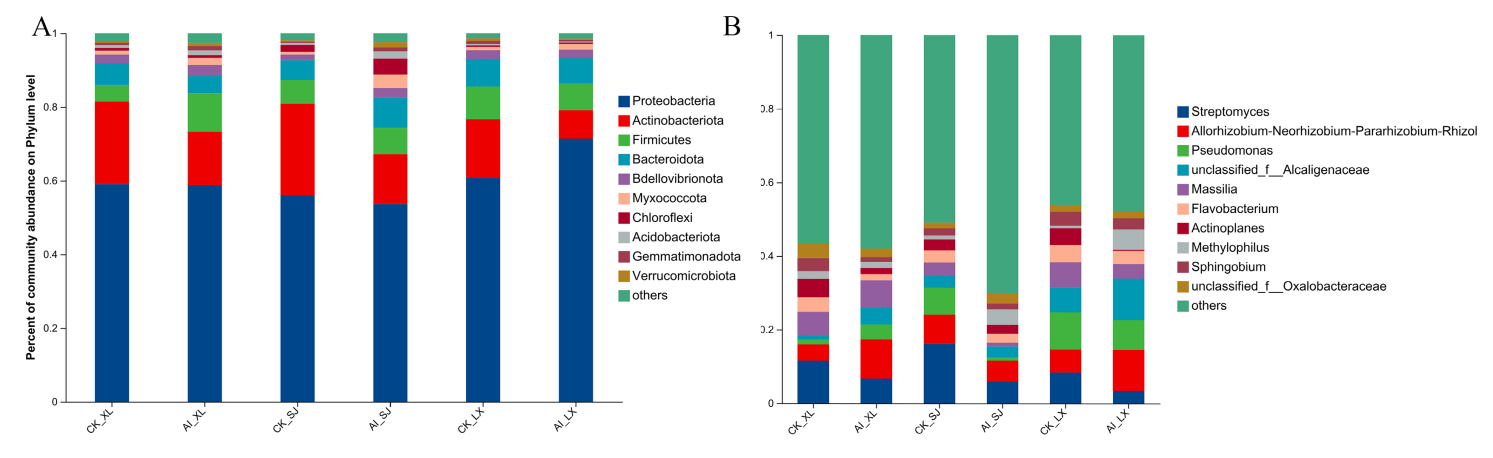


**Supplementary Figure 2.** The figure legends are required to have the same font as the main text, 12 point normal Times New Roman, single spaced. Please use a single paragraph for each legend and prepare the figures keeping in mind the PDF layout.


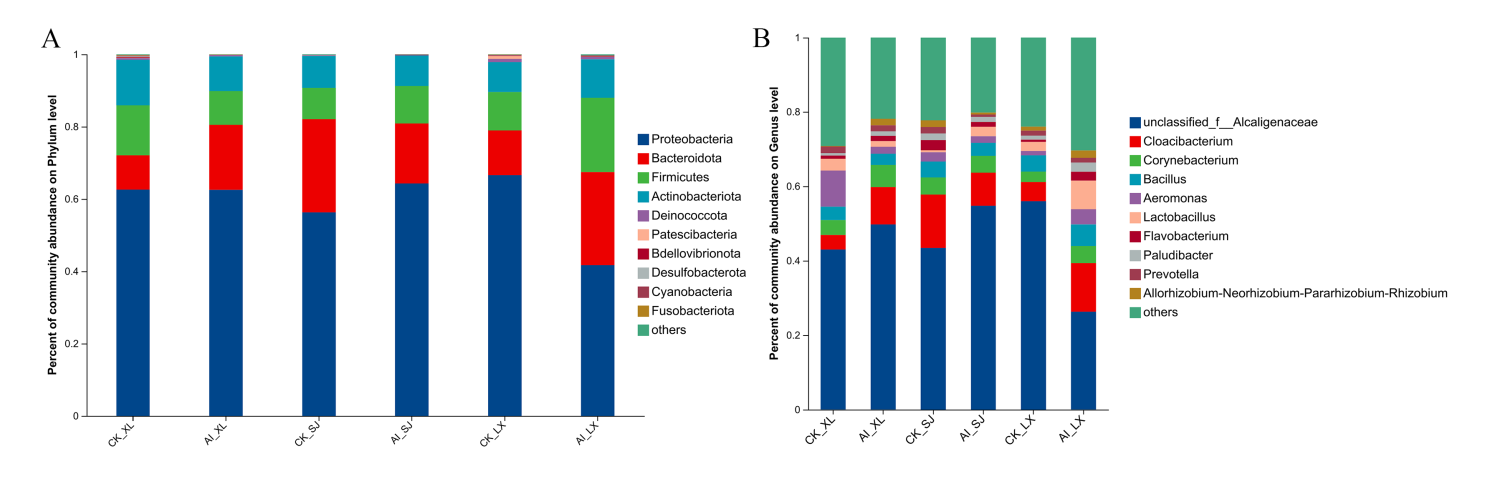


**Supplementary Figure 3.** The figure legends are required to have the same font as the main text, 12 point normal Times New Roman, single spaced. Please use a single paragraph for each legend and prepare the figures keeping in mind the PDF layout.

**
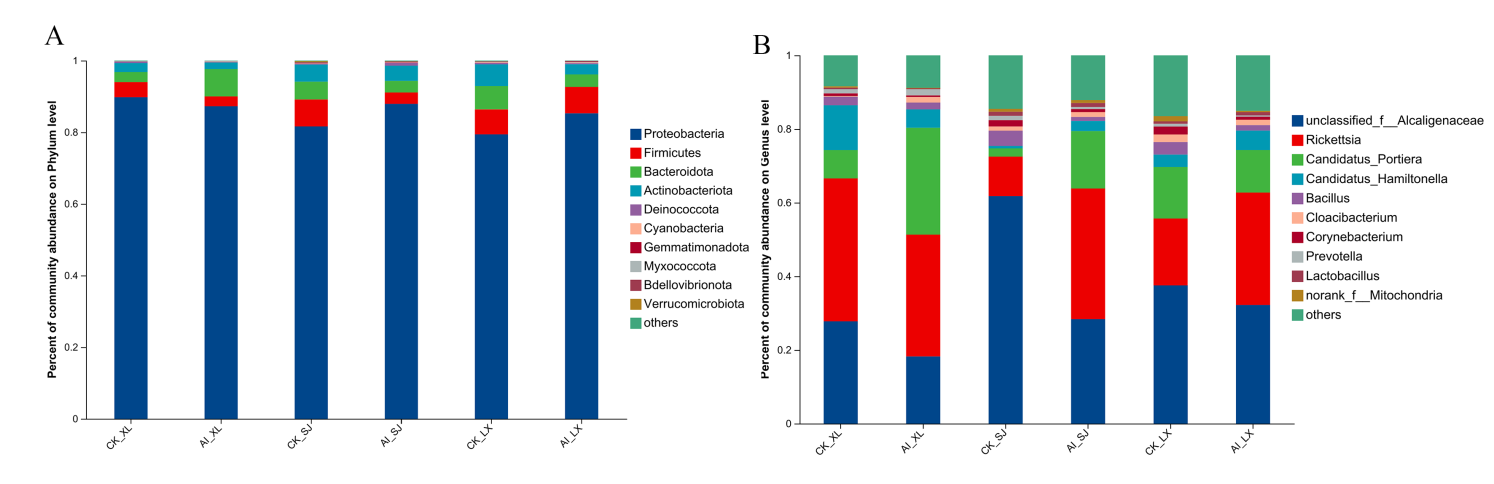
**

**Supplementary Figure 4.** The figure legends are required to have the same font as the main text, 12 point normal Times New Roman, single spaced. Please use a single paragraph for each legend and prepare the figures keeping in mind the PDF layout.
